# Dependence of Atomic Thickness on Interfacial Conditions and Magnetocrystalline Anisotropy in SmCo_5_/Sm_2_Co_17_ Multilayer

**DOI:** 10.3390/ma12010056

**Published:** 2018-12-24

**Authors:** Soyoung Jekal

**Affiliations:** 1 Laboratory of Metal Physics and Technology, Department of Materials, ETH Zurich, 8093 Zurich, Switzerland; so-young.jekal@mat.ethz.ch; Tel.: +41-44-632-26-43; 2 Condensed Matter Theory Group, Paul Scherrer Institute, CH-5232 Villigen PSI, Switzerland

**Keywords:** first principles, exchange energy, magnetocrystalline anisotropy

## Abstract

We have performed first-principles calculations to study the interfacial exchange coupling and magnetocrystalline anisotropy energy in a SmCo${}_{5}$/Sm${}_{2}$Co${}_{17}$ multilayer model system. The phase of SmCo${}_{5}$ and Sm${}_{2}$Co${}_{17}$ stacking along (0001) direction are structurally well matched. The atomic structure, including the alignment and the separation between layers, were firstly optimized. Then the non-collinear magnetic structures were calculated to explore the exchange coupling across the interface and the variation of magnetocrystalline anisotropy energy. We found that the inter-phase exchange coupling strength, rotating behavior and magnetocrystalline anisotropy strongly depend on the atomic thickness of the SmCo${}_{5}$ and Sm${}_{2}$Co${}_{17}$ phase.

## 1. Introduction

Since Kneller and Hawig’s pioneering work [1] on composite permanent magnetic materials consisting of a mixture of hard and soft magnetic phases, exchange coupled permanent magnets have been extensively studied to achieve high maximum energy product values [2,3,4,5]. Interestingly, shape of their demagnetization curves is similar with that of typical single-phase materials, although these materials consist of two ferromagnetic phases, at least [6,7]. By taking into account exchange coupling between the small grains of the magnetic phases, the single-phase behavior and the remanence enhancement have been well understood [1]. So far, many remanence-enhanced magnets based on nanocrystalline mixtures of the hard and soft phases have been found [8,9,10], and the maximum energy product values have been expected to be enhanced in these hard/soft composite systems, by combining large magnetic anisotropy of a hard phase and high saturation magnetization of a soft phase [11].

Among the commonly used magnetic materials, SmCo${}_{5}$ has the largest magnetocrystalline anisotropy energy of 17.2 MJ/m${}^{3}$ with high Curie temperature of about 1000 K, while 3*d*-transition metals such as Fe, Co, Ni and their alloys have very high Curie temperatures with large saturation magnetizations [12,13,14,15]. Not only in magnetically hard/soft phases but also in the mixtures of two magnetically hard phases, SmCo${}_{5}$ and Sm${}_{2}$Co${}_{17}$, the spring-magnet behavior and exchange coupling were observed similar with hard/soft composite system. Since Sm${}_{2}$Co${}_{17}$ has both a high Curie temperature and a high magnetocrystalline anisotropy [16,17,18,19], it is an important material system to overcome magnetically hard/soft mixtures.

According to early models by Kneller [1] an ideal hard/soft phase multilayer achieves maximum energy product at the optimum thickness of the soft phase which is equal to two domain wall thickness in the hard phase (~7 nm for SmCo${}_{5}$). However, many recent experimental and theoretical studies show the important effect of the soft phase parameters and interface conditions [20,21,22,23,24]. Thus it is important to understand the influence of these factors in the inter-phase exchange coupling, in order to achieve better energy products. These issues can be tackled in the scope of first-principles electronic structure calculations based on density functional theory (DFT) as demonstrated in previous works [25,26].

In the present work, we focused on the effects of the atomic thickness on the interface conditions and the magnetocrystalline anisotropy energy in layered SmCo${}_{5}$/Sm${}_{2}$Co${}_{17}$ system using first-principles methods. We show that magnetocrystalline anisotropy and exchange coupling on the interface between the hard (SmCo${}_{5}$) and soft (Sm${}_{2}$Co${}_{17}$) phase can be modified by atomic thickness of SmCo${}_{5}$ and Sm${}_{2}$Co${}_{17}$ layers.

## 2. Methods

DFT calculations were performed on the multilayer of the SmCo${}_{5}$/Sm${}_{2}$Co${}_{17}$ with various atomic thickness using plane-wave basis sets and pseudopotentials, implemented in Quantum Espresso (QE) [27], which enables time-efficient calculations. For the exchange-correlation potential we adapted the local spin-density approximation plus Hubbard *U* (LSDA + *U*), which can adequately describe the strongly correlated electronic states of 4*f* electrons [28,29,30]. Since the LSDA + *U* method requires the Coulomb energy (*U*) and the exchange energy (J) as input parameters, *U* and J were defined through the derivatives of the energy levels $\epsilon_{f}$ of the *f*-orbital with respect to their occupancies $n_{f}$, described as $U=\partial \epsilon_{f↑}/\partial n_{f↓}$ and $J=\partial \left(\epsilon_{f↑}-\epsilon_{f↓}\right)/\partial \left(n_{f↑}-n_{f↓}\right)$ for the majority (minority) spin ↑ (↓), respectively. From these expressions we obtain U=6.0 eV and J=1.0 eV for Sm.

The wave functions were expanded by a plane-wave basis set with an optimized cutoff energy of 340 Ry, and the Brillouin zone was sampled via a 12×12×4 *k*-point mesh. Different mesh values from 72 to 980 were tested to ensure the precise of our calculations, with the convergence criterion being 0.1 μeV. The convergence with respect to cutoff was also carefully checked.

### 2.1. Atomic Structure

**Figure 1:**
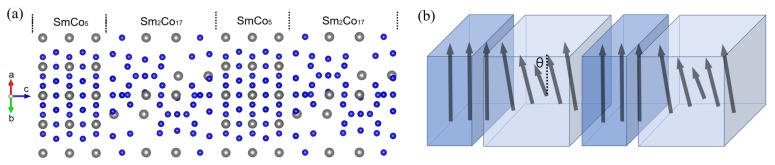
(**a**) Atomic configurations of the two phase multilayered system. The 5-monolayer (ML) of hard (SmCo${}_{5}$) and 7-ML of soft (Sm${}_{2}$Co${}_{17}$) phases are aligned along (0001) direction. The gray large, blue small balls represent Sm and Co atoms, respectively. The dotted lines on top of panel indicate the interface between two phases. The periodic boundary condition has been used; (**b**) A schematic diagram of non-collinear magnetic orderings in the systems. The arrows represent the directions of magnetic moments of the atoms in each layer. By total energy calculation, it is confirmed that spin prefers to rotate along in-plane (parallel to interface). θ is the angle between the directions of magnetic moments of the atoms in the hard phase and those in the middle layer of the soft phase, which are fixed.

The multilayered model consists of SmCo${}_{5}$ and Sm${}_{2}$Co${}_{17}$ layers stacking along (0001) direction is adopted to construct the interface structure for our simulation as shown in Figure 1a. Since the lattice constants of SmCo${}_{5}$ and Sm${}_{2}$Co${}_{17}$ have a mismatch of 17% along this direction, the self-consistent spin-polarized electronic structure calculations with periodic boundary conditions was carried out with fully relaxation to optimize the atomic structure.

### 2.2. Exchange Coupling

The exchange-spring multilayer with the size of the hard and soft layer smaller than the thickness of a usual domain wall is considered, therefore, the exchange-coupling between the two phases will be in effect. A single domain case is considered for both the hard and the soft phase in the present modeling interface. To describe the exchange coupling strength between the soft and hard phases, we model a simulated demagnetization process of the magnetic systems using non-collinear magnetic structure calculations. In this method the direction of local magnetic moments at the center of soft layer is rotated from a ferromagnetic state to a finite angle. We vary this angle as a parameter to extract the strength of the interlayer exchange coupling. This result is double-checked by using a perturbative method.

### 2.3. Magnetocrystalline Anisotropy Energy

The magnetocrystalline anisotropy energy was calculated using the force theorem. It is defined as the total energy difference between the magnetization perpendicular to the [1000]-plane and parallel to the [1000]-plane, i.e., $K=E_{\left[1000\right]}-E_{\left[0001\right]}$, where $E_{\left[1000\right]}$ and $E_{\left[0001\right]}$ are the total energies with the magnetization aligned along the hard- ([1000]) and easy-axis ([0001]) of the magnetic anisotropy, respectively. Specifically, magnetocrystalline anisotropy energy is calculated in three steps: first, collinear self-consistent calculation is performed without spin-orbit coupling; second, the density matrix is globally rotated to consider the magnetization along [1000] and [0001] to calculate $E_{\left[1000\right]}$ and $E_{\left[0001\right]}$; and finally, non-collinear and non-self-consistent calculation is performed with spin-orbit coupling.

## 3. Results

### 3.1. Atomic Structures & Magnectic Moments

**Figure 2:**
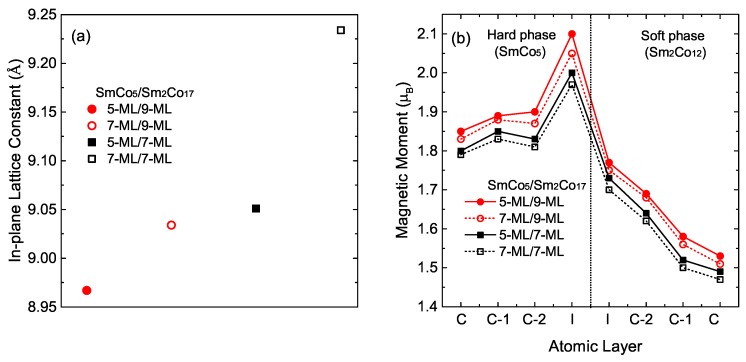
(**a**) The optimized in-plane lattice constant of the multilayers with different number of atomic monolayer (ML); (**b**) Calculated average magnetic moment of Co atoms with respect to the atomic layer. While C and C-n denote the centered layer and the n layer below the center, respectively, I denotes the interface between SmCo${}_{5}$ and Sm${}_{2}$Co${}_{17}$.

We firstly found the equilibrium structural parameters of considered multilayers. Atoms were fully relaxed along c-direction while the in-plane lattice parameter a${}_{in}$ is fixed to 8.367 Å ≤ a ≤ 9.834 Å. In the optimized structures, we investigated average magnetic moments of each layers of multilayered system which consist different thickness of hard and soft phases. Figure 2a,b present calculated in-plane lattice constants and magnetic moments. Since in-plance lattice of (2 × 2) SmCo${}_{5}$ is 17% larger than (1 × 1) Sm${}_{2}$Co${}_{17}$, in-plane lattice constant is getting increase with thicker SmCo${}_{5}$ and thinner Sm${}_{2}$Co${}_{17}$, as expected. Regardless of atomic thickness, interface atoms between soft and hard phase show noticeably large magnetic moments compared to the that of centered atoms. The magnetic moment gradually decreased from the interface to the center, especially in the soft phase.

### 3.2. Exchange Energies

**Figure 3:**
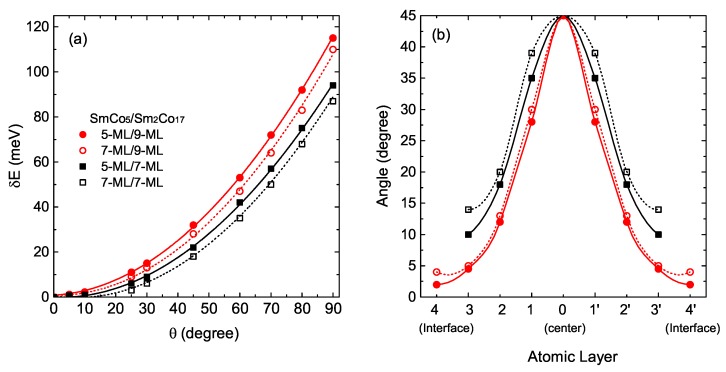
(**a**) The calculated total energy difference, δE(θ) = E(θ) − E(θ = 0${}^{\circ }$) and their fitting to a quadratic curve for the four systems with various atomic thickness; (**b**) The angle distributions for the soft phase atomic layers parallel to the interface plane (refer to Figure 1). 0 label represent centered layer which is the middle layer of the soft phase, whose atomic magnetic moments are turned at a fixed value of 45${}^{\circ }$ away from those in the hard phase layers. All the atomic magnetic moment orientation in layers 1 (1${}^{\prime }$), 2 (2${}^{\prime }$), 3 (3${}^{\prime }$), and 4 (4${}^{\prime }$) are obtained self-consistently.

The nature of the magnetic reversal processes is an important issue for assessing the applicability of exchange-spring magnets. To address this problem, the optimized structure is used as an input for non-collinear magnetic order calculations using The full-potential linear muffin-tin orbital (FP-LMTO) method [31]. Our model mimics a domain wall which forms in the demagnetization process. We consider the directions of the magnetic moments of the atoms in the hard phase were fixed to the easy magnetization axis direction and seven or nine layers of soft phase with magnetic moments rotated from its ferromagnetic (FM) order as illustrated in Figure 1b. The magnetic moments of atoms in the middle layer of the soft phase were fixed to turn a given angle θ relative to the direction of the magnetic moments of the hard phase, while the magnetic moments of other atoms in the soft phase were free to relax. Upon the convergence of the calculations is reached, the total energy is obtained for each given angle θ. The total energy difference for the system, δE(θ) = E(θ) − E(θ = 0${}^{\circ }$), as a function of the turning angle θ is shown in Figure 3a.

We find that δE(θ) behaves as a quadratic function of θ, manifesting the spring behavior and the exchange coupling between the soft and hard phases in this system. We compare results in the case of the hard and soft phases made of different atomic thickness. The systems with thinner hard phase and thicker soft phase are expected to strengthen the exchange coupling. In Ref. [32], they demonstrated a reduced exchange energy at interface by showing the variation of the layer resolved angle of rotations of atomic moment across the soft phase. Exchange coupling energy is the strongest between the centered layers of soft phases, but weakest across the interface.

Interestingly, we find the considerable effect of atomic thickness of multilayers on the exchange coupling at interface. As shown in Figure 3, δE(θ) is a quadratic function of θ for whole systems. However, the curve of system with thinner hard phase and thicker soft phase is much steeper, indicating that the exchange coupling in this system is stronger than the other candidates. Comparing relaxed angles of magnetic moment, we observe smaller angle of rotation in the interface layer while angle of centered layers of soft phase is fixed as 45${}^{\circ }$.

To further understand the phenomena, we have calculated the site-to-site exchange interaction parameters $J_{ij}$ between sites *i* and *j*,

(1) Jij=14π×∫−∝EFdϵ∑m,m′,m″,m‴Im[Δimm′Gij↓m′m″(ϵ)Δjm″m‴Gij↑m‴m(ϵ)],

where $\Delta_{i}^{mm^{\prime }}$=$\int_{\mathrm{BZ}}$[$H_{ii↑}^{mm^{\prime }}$(*k*)-$H_{ii↓}^{mm^{\prime }}$(*k*)]d*k* is the exchange splitting within the Brillouin zone and $G_{ij↓}^{m^{\prime }m^{\prime \prime }}$(ϵ) is the real-space Green’s function [33].

Since $J_{ij}$ decreases rapidly as a function of the distance, the calculation is limited to the few nearest neighboring pairs only. The $J_{ij}$ for pairs across the hard/soft interface are averaged over the atomic pairs between the two layers and the results for the four models of 5-ML/9-ML, 7-ML/9-ML, 5-ML/7-ML, and 7-ML/7-ML, are 130.01, 129.83, 124.76, 122.45 meV, respectively, in the ferromagnetic state. It is clear that the absolute values of site-to-site exchange parameters of the interface atomic pairs in system 5-ML(SmCo${}_{5}$)/9-ML(Sm${}_{2}$Co${}_{17}$) are larger than those of the corresponding pairs in other candidates we considered. This also supports that the inter-phase exchange coupling in system with thinner hard phase and thicker soft phase is stronger, in agreement with the present non-collinear magnetic order calculation as discussed above.

### 3.3. Magnetocrystalline Anisotropy

We also find a atomic thickness dependent magnetocrystalline anisotropy in SmCo${}_{5}$/Sm${}_{2}$Co${}_{17}$ multilayers. In fact, for 5-ML/9-ML the anisotropy energy is ~2.3 meV/atom, which is 35% larger than that of bulk SmCo${}_{5}$ (~1.5 meV/atom) [14]. However, it decrease in the systems with thicker hard phase or thinner soft phase, due to the increased symmetry.

**Figure 4:**
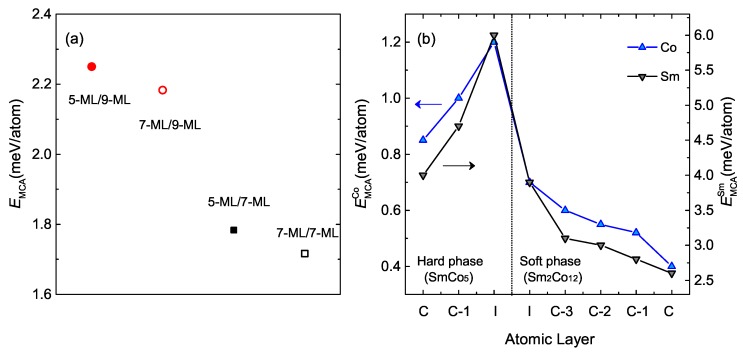
(**a**) Magnetocrystalline anisotropy energy ($E_{\mathrm{MCA}}$); (**b**) Partial $E_{\mathrm{MCA}}$ of Co and Sm with respect to the atomic layer of 5-ML/9-ML multilayer.

Even if this system is a multilayer with broken symmetry unlike a bulk SmCo${}_{5}$, the 35% enhancement in magnetocrystalline anisotropy energy for 5-ML/9-ML is unexpected. As shown in Figure 4b, the main contribution to the enhancement of magnetocrystalline anisotropy energy comes from the interface between hard and soft phases. The origin of large magnetocrystalline anisotropy is ascribed to the spin-orbit induced mixing between 4*f* and 3*d* orbitals at the interface between the hard and soft phases.

## 4. Conclusions

We have carried out first-principles calculations of the magnetocrystalline anisotropy energy and the exchange coupling across SmCo${}_{5}$/Sm${}_{2}$Co${}_{17}$ multilayers. Using both the non-collinear magnetic structure simulation and the calculation of the site-to-site exchange parameters across the interfaces, we found that the exchange coupling in SmCo${}_{5}$/Sm${}_{2}$Co${}_{17}$ is enhanced by the thin (<5-ML) hard phase and thick (>9-ML) soft phase. This system also shows the strongest magnetocrystalline anisotropy energy of 2.25 meV/atom among other candidates we considered, and the most contribution comes from the interface between hard and soft phases. The origin of large magnetocrystalline anisotropy is ascribed to the spin-orbit induced mixing between 4*f* and 3*d* orbitals at the interface between the hard and soft phases.
